# RNAi-based bioinsecticide for *Aedes* mosquito control

**DOI:** 10.1038/s41598-019-39666-5

**Published:** 2019-03-11

**Authors:** Sheila Barbara G. Lopez, Victor Guimarães-Ribeiro, João Victor G. Rodriguez, Fernando A. P. S. Dorand, Tiago S. Salles, Thayane E. Sá-Guimarães, Evelyn S. L. Alvarenga, Ana Claudia A. Melo, Rodrigo V. Almeida, Monica F. Moreira

**Affiliations:** 1 Universidade Federal do Rio de Janeiro, Departamento de Bioquímica, Instituto de Química, 21941-909 Rio de Janeiro, RJ Brazil; 2 Instituto Nacional de Ciência e Tecnologia em Entomologia Molecular, Rio de Janeiro, RJ Brazil

## Abstract

Zika virus infection and dengue and chikungunya fevers are emerging viral diseases that have become public health threats. Their aetiologic agents are transmitted by the bite of genus *Aedes* mosquitoes. Without effective therapies or vaccines, vector control is the main strategy for preventing the spread of these diseases. Increased insecticide resistance calls for biorational actions focused on control of the target vector population. The chitin required for larval survival structures is a good target for biorational control. Chitin synthases A and B (CHS) are enzymes in the chitin synthesis pathway. Double-stranded RNA (dsRNA)-mediated gene silencing (RNAi) achieves specific knockdown of target proteins. Our goal in this work, a new proposed RNAi-based bioinsecticide, was developed as a potential strategy for mosquito population control. DsRNA molecules that target five different regions in the CHSA and B transcript sequences were produced *in vitro* and *in vivo* through expression in *E*. *coli* HT115 and tested by direct addition to larval breeding water. Mature and immature larvae treated with dsRNA targeting CHS catalytic sites showed significantly decreased viability associated with a reduction in CHS transcript levels. The few larval and adult survivors displayed an altered morphology and chitin content. In association with diflubenzuron, this bioinsecticide exhibited insecticidal adjuvant properties.

## Introduction

Arboviruses, such as Yellow fever virus, Dengue virus serotypes 1–4^1,2^, Zika virus and Chikungunya virus^3^, are responsible for significant human morbidity and mortality in affected regions. These diseases are transmitted by mosquito bites and have an enormous impact on public health. The distribution of dengue virus has increased dramatically, although actual numbers of dengue cases have been underreported because many are misclassified. One recent estimate indicated 390 million dengue infections occur per year worldwide, of which 96 million manifest clinically as a disease with varying severity^4^. On the American continent, a total of approximately of 577,697 dengue cases were reported, including 4,366 severe dengue cases^2^. Yellow fever is a severe disease that affects many countries. In Brazil, from December 2016 to July 2017, the Brazilian Ministry of Health (MH) reported 3,564 cases of yellow fever^5^. Zika virus, belonging to *Flaviviridae*, causes a fever disease similar to dengue, but connections of Zika virus with neonatal microcephaly and Guillain-Barré syndrome have also been demonstrated recently^6^. A member of the family *Togaviridae*, Chikungunya virus, causes a febrile disease characterized by severe chronic arthralgias^7^.

The major vector of these diseases, the *Aedes aegypti* (*Ae*. *aegypti*) mosquito (Linnaeus, 1762), has opportunistic habits and lives in or near residences. The mosquito life cycle comprises four stages: egg, larva (L1–4), pupa and adult. When eggs encounter water, the larvae hatch after a few hours, pass through four instars of development within a period of approximately seven days, and then enter the pupal stage. The pupae then undergo metamorphosis and give rise to the winged adult form, which is reproductively mature and capable of transmitting diseases during the consumption of its blood meal.

Several different species of mosquitoes exhibit insecticide resistance at alarming rates^8,9^. The short life cycle and abundant offspring of insects favour the emergence of populations with different genetic characteristics, priming insect populations for insecticide resistance. Insecticide resistance is then caused by frequent insecticide use, as a result of selective pressure on different hereditary populations^10^. Therefore, alternative strategies for vector control have been developed, such as the use of predatory fishes and fungi, to reduce vector dissemination^11^. *Bacillus thuringiensis* toxin^12–14^ and *Wolbachia*^15^-infected mosquito dissemination have been shown to be effective for mosquito control; however, the long-term effectiveness of these methods remains to be accurately evaluated^11^.

Chitin, an N-acetylglucosamine polymer, is the major constituent of important extracellular barriers, such as the exoskeleton (cuticle), the peritrophic matrix (PM) in the intestine^16,17^ and the eggshell^18^, which protect insects and ensure their survival in the environment. The last step of the chitin biosynthesis pathway is executed by the chitin synthase protein (CHS), which belongs to the family of glycosyltransferase enzymes^19^. Insects commonly possess two chitin synthase genes: *CHS1*, or *CHSA*, is primarily involved in chitin synthesis for the exoskeleton cuticle^17,19^ and eggs^20,21,22^, while *CHS2*, or *CHSB*, is responsible for chitin synthesis in the intestinal PM^16,19,23,24^. Due to the roles of chitin in all stages of the mosquito’s life, chitin is a good control target, allowing intervention at different stages of development by altering the formation of the cuticle and PM in the larval and adult stages or in eggs.

In mosquitoes, the mechanism of RNAi has been widely used to study gene function, and its potential has been explored as a new alternative for population control^25^. “For effective RNAi in insects, the dsRNA has to function inside the cells. At least two types of dsRNA uptake mechanism have been proposed so far: transmembrane-channel-mediated uptake and endocytosis-mediated uptake”^25–27^. In the target cell, the RNAi machinery processes dsRNAs, which are cleaved by the RNase III-*Dicer* enzyme into 20–25-nucleotide small interfering RNAs (siRNAs). Subsequently, Argonaute proteins assemble this siRNA to form an RNA-induced silencing complex (RISC) that targets the destruction of the endogenous mRNA complementary to its guide strand^28,29^. As dsRNA uptake is the main bottleneck to vector or pest control by RNAi, simpler methods of dsRNA administration, such as soaking^30^, oral feeding^31,32^ and micro-sprayer use^33^, have opened up the possibility of exploring the use of dsRNA as a biopesticide or bioinsecticide^8^. Furthermore, the specificity of RNAi makes these approaches environmentally safer than other methods currently in use, minimizing toxicity to non-target species and reducing the possibility of resistance in insect populations^33–35^.

In this work, we propose a low-cost approach for producing a potential bioinsecticide that could be applied via direct addition to breeding water, without a carrier. The bioproduct, based on lysates of recombinant *E*. *coli* expressing dsRNA, caused a significant mortality rate, even in the treatment of 4^th^-instar larva, which was associated with a reduction in CHSA and B transcripts and, consequently, decreased chitin content in the PM and cuticle. Additionally, this approach was shown to exert an insecticidal adjuvant effect in association with the insecticide diflubenzuron (DFB), a chitin synthesis inhibitor.

## Results

### Protein and nucleotide sequence searches

Blast analysis of the *Ae*. *aegypti* protein database allowed the identification of two proteins with high identity to the *Tribolium castaneum* CHSA (NP_0010394021) query sequence: AAEL002718-PA, CHSA, and AAEL005618-PA, CHSB (data not shown). The homologous nucleotide sequences of the putative CHS genes AAEL002718-RA (CHSA) and AAEL005618-RA (CHSB) were aligned as described in Material and Methods (Suppl Fig. 1). In the CHS nucleotide alignment, the regions chosen as targets for gene silencing were highlighted: CHSA 2718_1 (1550–1750 nt), 2718_2 (1064–1291 nt), 2718_3 (1928–2014 nt), CHSB 5618_1 (1205–1384 nt) and 5618_2 (693–940 nt) (Table 1).

**Table 1:** Target regions for posttranscriptional silencing of CHS A (2718) and CHS B (5618) and the sequences of primers used for double-stranded RNA (dsRNA) synthesis and quantitative PCR (qPCR).

Target regions for posttranscriptional silencing	Oligonucleotides for silencing
2718_1	F_5′TAATACGACTCACTATAGGGCCTATTTTCGTTGTTGTTCGGAG3′ R_5′TAATACGACTCACTATAGGGCCGAAAACGTAGCACAAGTA3′
2718_2	F_5′TAATACGACTAACTATAGGGACACTTTTTGCTCGTGTTC3′ R_5′TAATACGACTCACTATAGGGAAGCCGGTAATTTGCGCAG3′
5618_1	F_5′TAATACGACTCACTATAGGGTTTCGCCATGTTCAGCAACG3′ R_5′TAATACGACTCACTATAGGGTGGGCACTGATGCCA3′
5618_2	F_5′TAATACGACTCACTATAGGGTTCTTCAAGAACATTCCAC3′ R_5′TAATACGACTCACTATAGGGTGGGCACTGATGCCA3′
2718_3	F_5′TAATACGACTCACTATAGGGGTTAAGTGATTTTGCATCA3′ R_5′TAATACGACTCACTATAGGGACGTCTCCTGTTTCAGCGCC3′
**qPCR**	**Oligonucleotides for verifying gene silencing**
CHSA	F_5′TCGTTGTTCGGAGTTGGGTT 3′ R_5′CTGAATGATCAAAACATACG 3′
CHSB	F_5′CCATGTTCAGCAACGGCT 3′ R_5′AGAACGTGTGTCACCGTAAT 3′

### Larvicidal effect assays using *in vitro*-synthesized dsRNA

**Figure 1:**
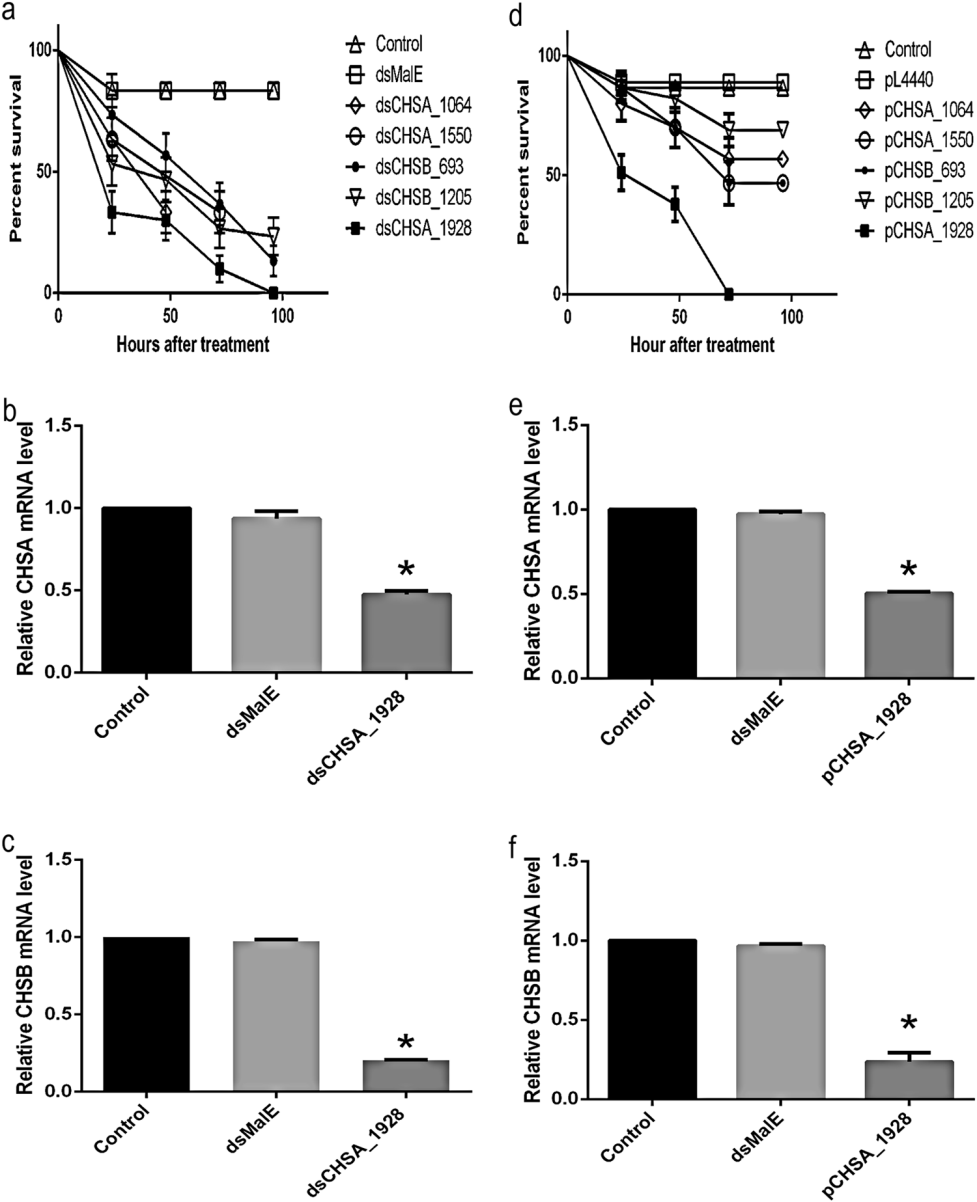
Survival curve of all experimental groups of larvae (1^st^-instar) treated with 0.2 µg/mL of each purified dsRNA (400 ng dsRNA/2 mL) (**a**) and treated with 2 × 10^−2^ µg/mL of *E*. *coli* HT115 lysate expressing each dsRNA (~4000 ng dsRNA/2 mL) from the PL4440 plasmid: pCHSA_1064 (dsCHSA_1064), pCHSA_1550 (dsCHSA_1550), pCHSB_693 (dsCHSB_693), pCHSB_1205 (dsCHSB_1205) and pCHSA_1928 (dsCHSA_1928) (**d**); both treatments target different regions in CHSA and B: dsRNA2718_1 (dsCHSA_1064), dsRNA2718_2 (dsCHSA_1550), dsRNA5618_1 (dsCHSB_693), dsRNA5618_2 (dsCHSB_1205) and dsRNA2718_3 (dsCHSA_1928). Relative expression of *CHSA* (**b**,**e**) and *CHSB* (**c**,**f**) transcripts in larvae (3^rd^-instar) after treatment. For the *in vitro* dsRNA experiment, the following controls were used: no addition of dsRNA or addition of dsRNA targeting the MalE gene, *E*. *coli* maltose-binding protein, an unrelated gene (dsMalE). As controls of the *E*. *coli* HT115 lysates expressing each dsRNA, the following treatments were used: PL4440 (*E*. *coli* HT115 lysate with empty PL4440 plasmid) or no dsRNA (control). The experiments were performed with three biological replicates. The RPS6 gene was used as an endogenous control to normalize the expression of CHS transcript levels. Bars represent the means ± SEM. All asterisks indicate significantly different values from those of the controls (ANOVA followed by Tukey’s test, P <0.05).

Naked dsCHSA_1064, dsCHSA_1550, dsCHSB_693, dsCHSB_1205 and dsCHSA_1928 synthesized *in vitro*, which target five different regions in CHSA and B, as described in Material and Methods, were added separately without a carrier to breeding water containing 1^st^-instar larvae. All five dsRNAs caused decreased survival rates during the experiment. However, a dramatic effect on the survival rate was observed at 24 h after treatment with dsCHSA_1928; this dsRNA targets a region, designated 2718_3, that includes fragments with continuous homology in both CHS sequences. The survival rate was approximately 95% in the control groups (without dsRNA and with dsMalE), and no significant difference was found between these groups (Fig. 1a). To confirm whether the large decrease in the larval survival rate found in the dsCHSA_1928 group was correlated with a reduction in CHSA and B transcript levels, a qPCR analysis was performed 72 h after dsRNA addition. This assay showed significant reductions in transcript levels for both CHSA and B, of approximately 50% and 80%, respectively (Fig. 1b,c), indicating that the decrease in the larval survival rate induced by dsCHSA_1928 was probably due to *CHSA* and *B* silencing.

### Larvicidal effect assays using *in vivo* dsRNA produced in the *E*. *coli* HT115 expression system

Five different clones, pCHSA_1064, pCHSA_1550, pCHSB_693, pCHSB_1205 and pCHSA_1928, were expressed *in vivo* using *E*. *coli* HT115 as an expression system, to induce dsCHSA_1064, dsCHSA_1550, dsCHSB_693, dsCHSB_1205 and dsCHSA_1928 production. Bacterial dsRNA expression after transformation with each recombinant PL4440 was induced with 1 mM IPTG or 10 g/L lactose. Larvicidal effects were observed in all groups as significant decreases in survival rates (Fig. 1b). In the control groups treated without dsRNA and with PL4440 (*E*. *coli* HT115 lysate with empty PL4440 plasmid), survival rates were not affected significantly. Accordingly, the highest impairment of the survival rates of 1^st^-instar larvae was observed 24 h after treatment in the larvae treated with the pCHSA_1928 bacterial lysate, containing dsCHSA_1928 (Fig. 1d). To further support the correlation between the larvicidal effects of pCHSA_1928 and *CHSA* and *B* silencing, qPCR was performed to measure the relative transcript levels of both CHS genes. The CHSA transcript level was reduced by approximately 50% (Fig. 1e), whereas the CHSB transcript level was reduced by approximately 80% (Fig. 1f). These data demonstrated that the significant decrease in larval survival observed after pCHSA_1928 treatment was probably associated with gene silencing. Therefore, the bacterial lysate suspension containing pCHSA_1928 was chosen as the bioinsecticide for subsequent experiments, due to its strong and rapid larvicidal effects.

### The bioinsecticide as adjuvant

**Figure 2:**
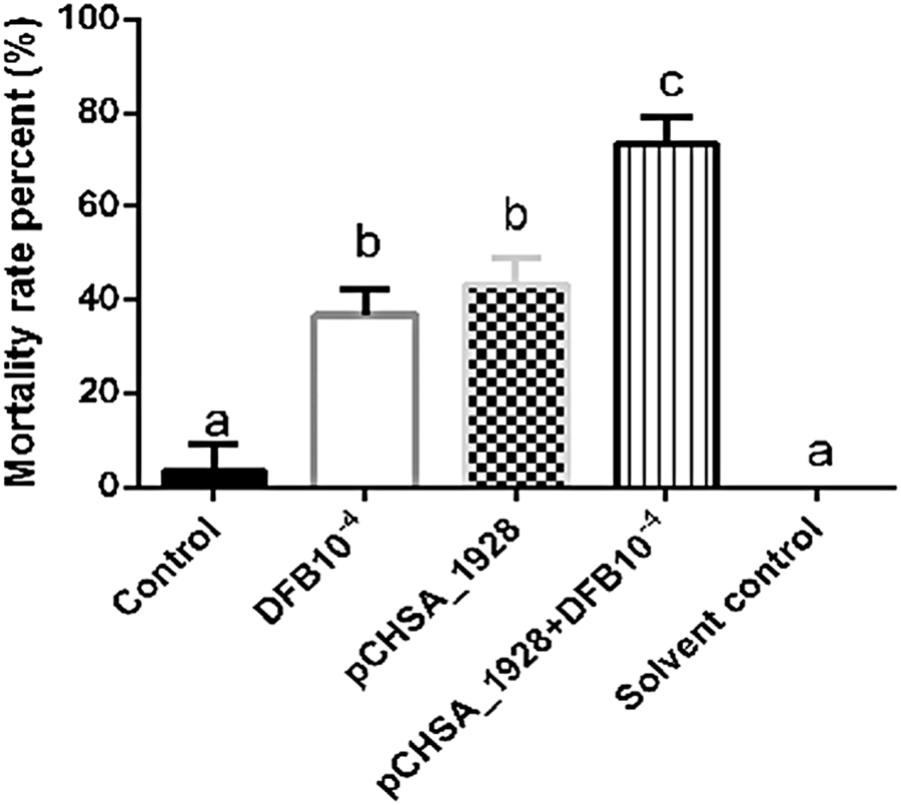
Insecticidal adjuvant effect of the bioinsecticide with the diflubenzuron insecticide (DFB) at a concentration of 10^−4^ mg/L (LC_50_) after six days of treatment. Control: not treated with bioinsecticide or DFB; DFB10^−4^ mg/L: 3^rd^-instar larvae treated with DFB 10^−4^ mg/L previously dissolved in acetone; pCHSA_1928: larvae treated with bioinsecticide (2 × 10^−2^ μg/mL); pCHSA_1928 + DFB10^−4^: larvae treated with 2 × 10^−2^ μg/mL bioinsecticide in the 1^st^-instar and DFB 10^−4^ mg/L in the 3^rd^-instar; Solvent control: larvae treated with acetone solvent at the same volume used in the DFB treatment groups.

To determine whether the insecticidal effect of DFB, a chitin inhibitor, could be improved by coadministration with a bioinsecticide, an experiment was performed combining both approaches in mosquito larvae. The larvicidal effect was measured on the 6^th^ day after treatment, and the results are presented as the percent mortality in Fig. 2. The larvae pre-treated with bioinsecticide exhibited an approximately 50% mortality rate on the 6^th^ day after treatment; when the larvae treated with 10^−4^ mg/L DFB reached the 3^rd^-instar, they exhibited an approximately 40% mortality rate on the same day; and when the larvae pre-treated with bioinsecticide and then exposed to 10^−4^ mg/L DFB reached the 3^rd^-instar, they exhibited a 75% mortality rate on the 6^th^ day after treatment. The control group treated with DFB solvent presented no mortality (Fig. 2). These results suggest that the bioinsecticide (pCHSA_1928) can act as an adjuvant that improves the performance of the insecticide DFB, possibly due to weakening insect chitinous structures.

### Larvicidal effect of the bioinsecticide in late stages of larval development

**Figure 3:**
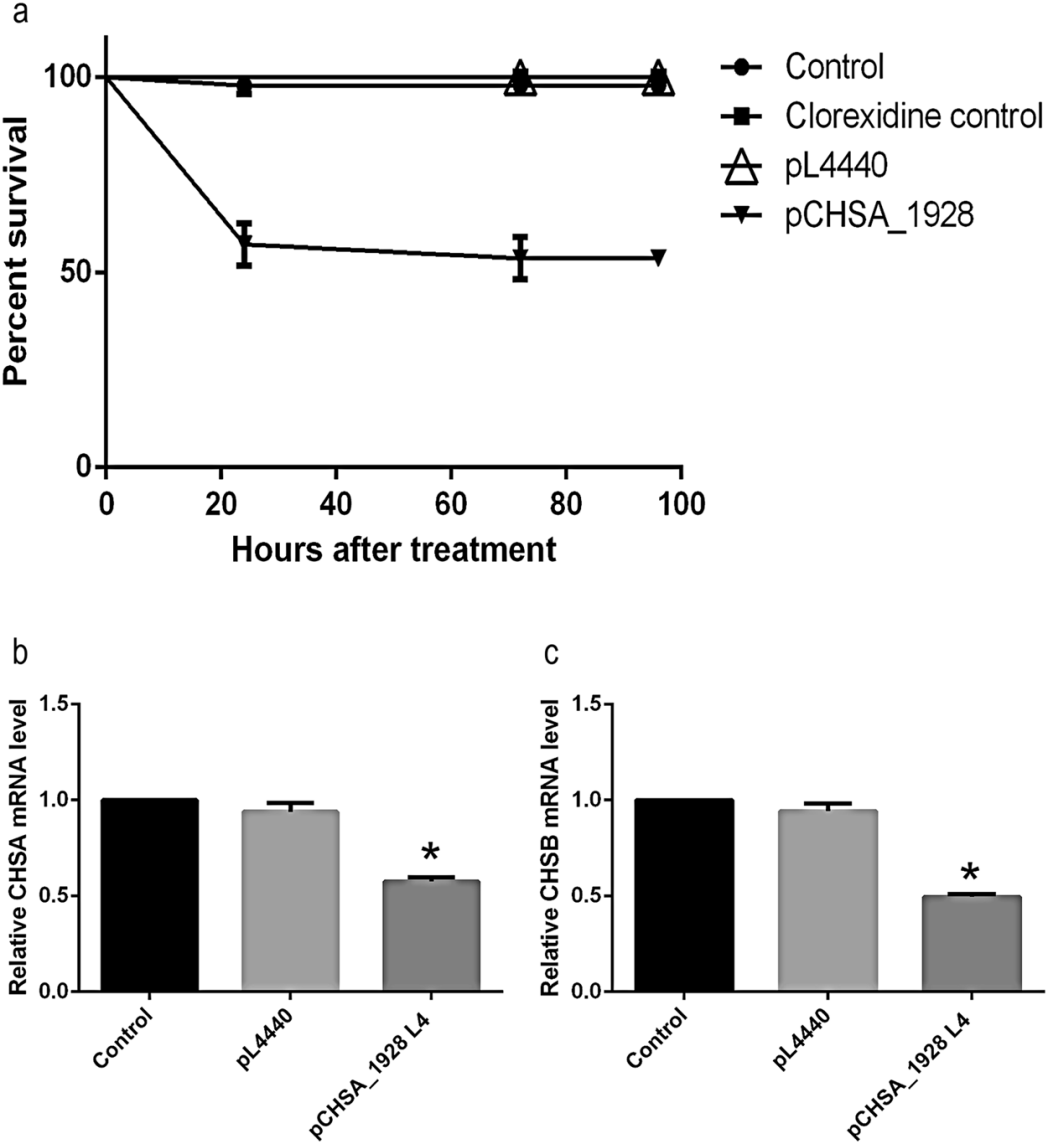
(**a**) Survival curves of experimental groups of larvae treated at the 4^th^-instar stage with 2 × 10^−2^ µg/mL of *E*. *coli* HT115 expressing dsCHSA_1928 lysed with chlorhexidine solution (bioinsecticide). Control groups: larvae without dsRNA addition (control), larvae with *E*. *coli* HT115 with empty PL4440 plasmid lysed with chlorhexidine (pL4440) and larvae exposed to *E*. *coli* HT115 lysed with chlorhexidine (chlorhexidine control). Relative expression of (**b**) *CHSA* and (**c**) *CHSB* in larvae treated at the 4^th^-instar with bacterial lysate expressing dsCHSA_1928 (pCHSA_1928), PL4440 (bacterial lysate with empty plasmid) or no dsRNA (control) (three biological replicates, three technical replicates). The RPS6 gene was used as an endogenous control to normalize the level of expression. Bars represent the means ± SEM. All asterisks indicate values significantly different from those of the controls (ANOVA followed by Tukey’s test, P < 0.05).

In the next step, the larvicidal effect of the bioinsecticide was tested in mature larvae. *E*. *coli* cells expressing dsCHSA_1928 were inactivated using chlorhexidine. As shown in Fig. 3a, 24 h after the addition of the bioinsecticide (2 × 10^−2^ µg/mL) to breeding water containing 4^th^-instar larvae, the survivorship rate was reduced by 60% relative to that in the control groups (larvae without dsRNA addition, larvae exposed to *E*. *coli* HT115 with empty PL4440 plasmid lysed with chlorhexidine and larvae exposed to *E*. *coli* HT115 lysed with chlorhexidine). The groups without bioinsecticide addition showed no significant reduction in the survival rate, and the larvae exhibited normal development during the observation time (data not shown). Even at an advanced stage of larval development, the bioinsecticide could reduce larval survivorship rates. To assess the effect of the bioinsecticide, the transcript levels of the *CHS* genes were also measured in the surviving mature larvae. Figure 3b shows a relative transcript level reduction in approximately 40% in CHSA, whereas relative CHSB expression was reduced by 50% (Fig. 3c). These reductions in CHSA and B transcript levels suggest that the decrease in survival rates in the bioinsecticide-treated groups can again be associated with gene silencing events.

**Figure 4:**
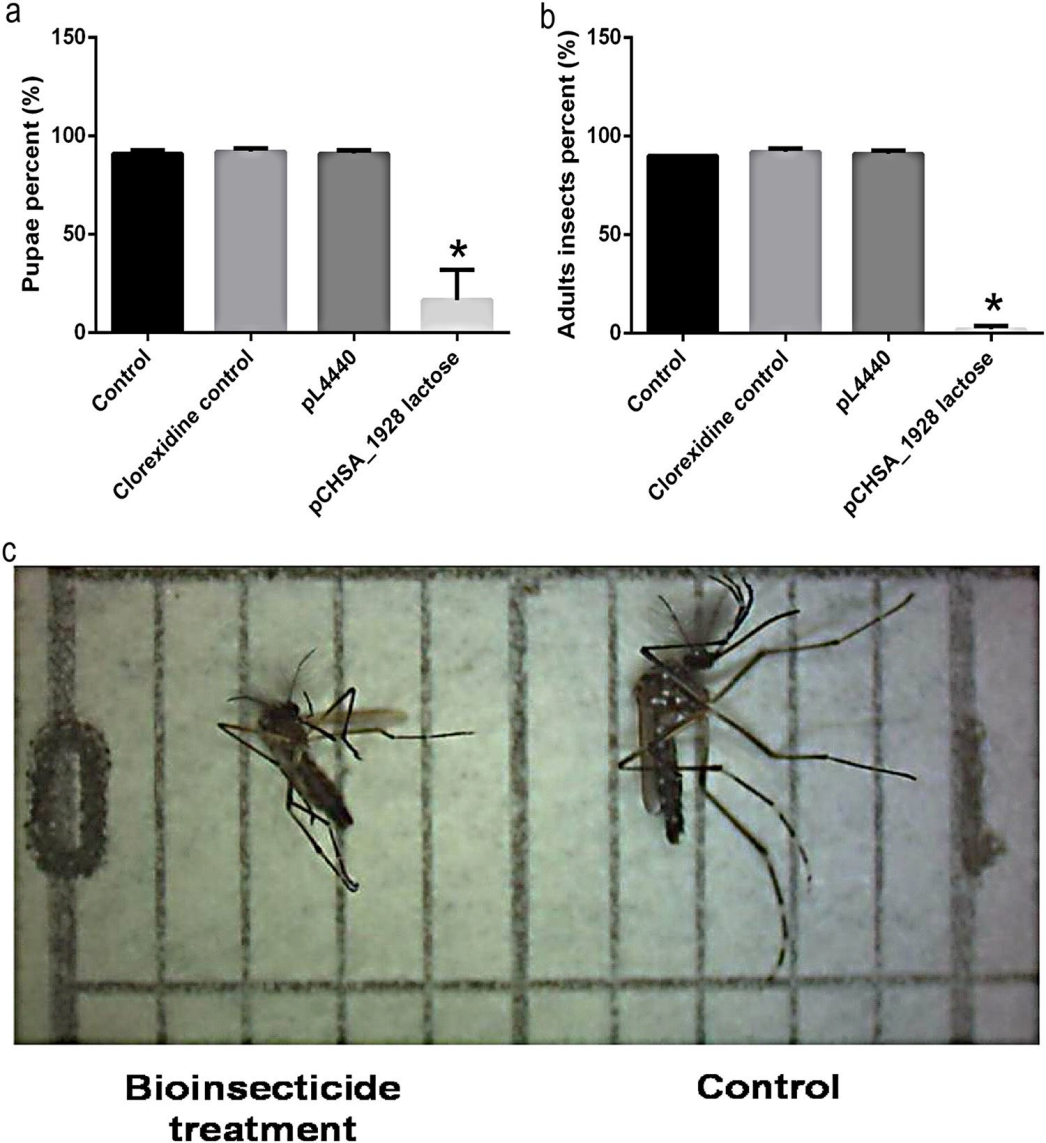
(**a**) Percentages of pupal moulting and (**b**) adult emergence of larvae treated at the 4^th^-instar with 2 × 10^−2^ µg/mL *E*. *coli* HT115 induced by lactose expressing dsCHSA_1928 lysed with chlorhexidine solution (bioinsecticide). Control groups: larvae with no dsRNA addition (control), to *E*. *coli* HT115 lysed with chlorhexidine at a concentration of 2 × 10^−2^ µg/mL. and larvae exposed to *E*. *coli* HT115 with empty PL4440 plasmid lysed with chlorhexidine (pL4440). Bars represent the means ± SEM. All asterisks indicate values significantly different from those of the controls (ANOVA followed by Tukey’s test, P < 0.05). (**c**) Male adult insects from larvae treated at the 4^th^-instar with *E*. *coli* HT115 expressing dsCHSA_1928 lysed with chlorhexidine solution (bioinsecticide treatment) and not treated with dsRNA (control). Each division on the scale is equivalent to 1 mm.

The 4^th^-instar larvae that survived after bioinsecticide treatment were tracked throughout their development, and it was observed that only 20% of the larvae treated with bioinsecticide developed into pupae (Fig. 4a). Larvae from the control groups without bioinsecticide addition developed normally. In each group of larvae treated with bioinsecticide in the 4^th^-instar, only one specimen reached the adult stage; the prevalence of this phenomenon was 3/45 of the total insects assayed (Fig. 4b). The control mosquitoes developed from eggs hatched at the same time as the bioinsecticide-treated ones and were collected for analysis at the same time as the treated ones; therefore, they were of the same age. Figure 4c shows that a surviving male insect (left) exhibited morphological alterations in the wings; these structures were abnormally reduced in size and fragile compared with those of a male insect from the control group, which exhibited normal development (right side). All these results demonstrated the larvicidal effects of bioinsecticide administration, even at later stages of larval development.

### The bioinsecticide still impacts larval survivorship at lower doses

**Figure 5:**
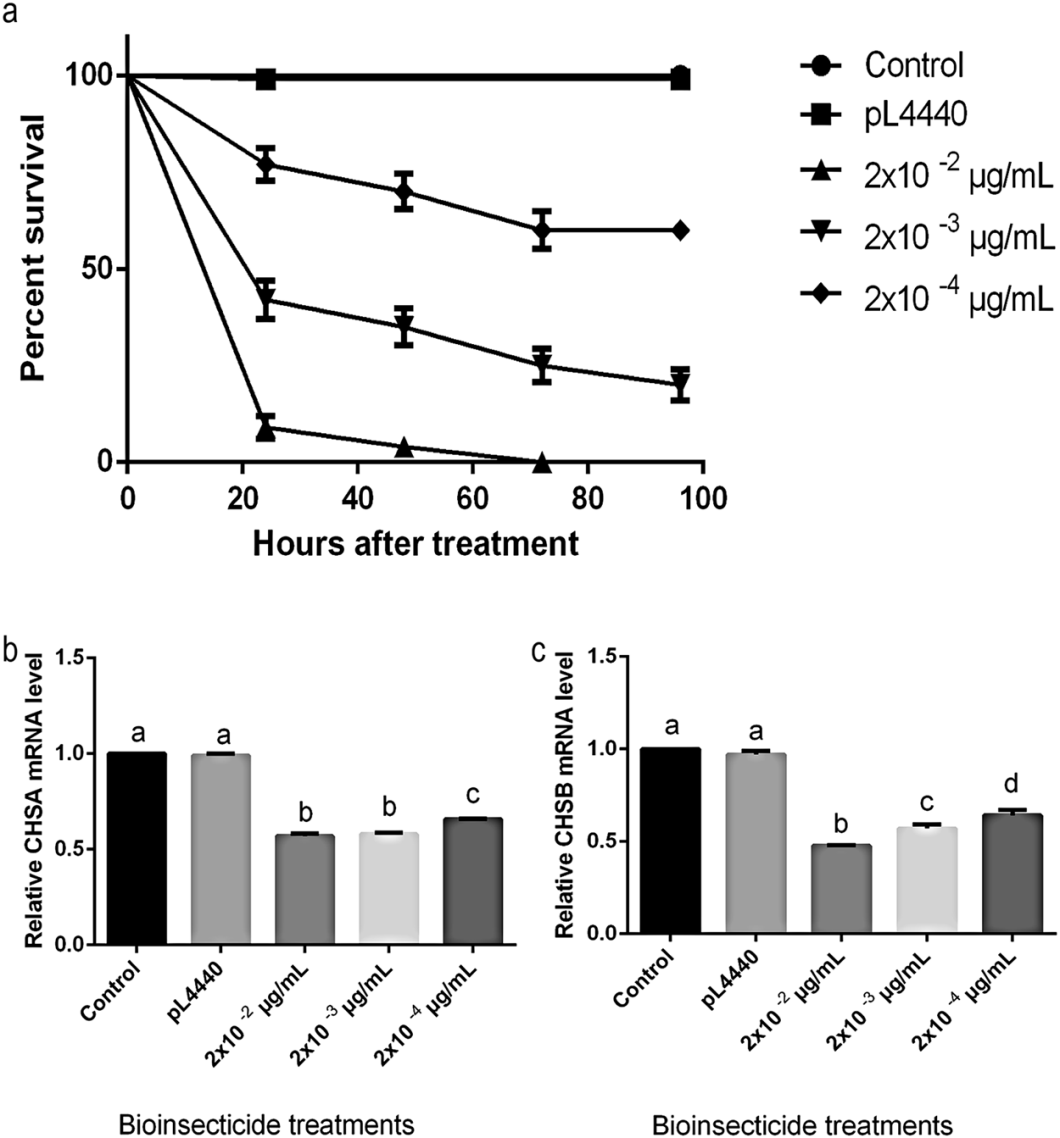
(**a**) Survival curve of experimental groups of larvae treated at the 1^st^-instar with different concentrations of bioinsecticide: 2 × 10^−2^ µg/mL, 2 × 10^−3^ µg/mL and 2 × 10^−4^ µg/mL. Control groups: larvae without dsRNA addition (control) and larvae with *E*. *coli* HT115 with empty PL4440 plasmid lysed with chlorhexidine (pL4440) (five biological replicates). Relative expression of (**b**) *CHSA* and (**c**) *CHSB* in larvae treated at the first instar with the three concentrations described above and untreated with dsRNA (control) (three biological replicates, five technical replicates). The *RPS6* gene was used as an endogenous control to normalize the level of expression. Bars represent the means ± SEM. All different letters indicate values significantly different from those of the controls (ANOVA followed by Tukey’s test, P < 0.05).

To determine whether lower doses of bioinsecticide treatment could also reduce larval survivorship, the bioinsecticide was administered at concentrations of 2 × 10^−2^ µg/mL and two orders of magnitude lower (2 × 10^−3^ and 2 × 10^−4^ µg/mL). The mortality rate was evaluated during 96 h after treatment. As control treatment, *E*. *coli* HT115 with empty PL4440 plasmid lysed with chlorhexidine was added directly to the rearing water of 1^st^-instar larvae and one group in the absence of dsRNA addition. As shown in Fig. 5a, the survival rate in the group treated with the highest dose (2 × 10^−2^ µg/mL) was reduced dramatically as soon as 24 h after bioinsecticide addition. Seventy-two hours after bioinsecticide treatment, no survivors remained in this experimental group. The experimental groups treated with 2 × 10^−4^ and 2 × 10^−3^ µg/mL of bioinsecticide exhibited survival reductions of approximately 60% and 25%, respectively, at the same time. The groups that were treated with the bioinsecticide at concentrations of 2 × 10^−2^, 2 × 10^−3^ and 2 × 10^−4^ µg/mL also showed decreases in CHSA transcript expression of approximately 60, 60 and 35%, respectively (Fig. 5b), whereas the expression of the CHSB transcript was reduced by approximately 50, 40 and 35% (Fig. 5c), respectively, compared to the control group without dsRNA treatment and the PL4440 group (*E*. *coli* HT115 lysate with empty PL4440 plasmid). This result showed the larvicidal efficacy of this bioinsecticide, even at concentrations that were lower by orders of magnitude. To determine LD50 and LD90 the data of survival rate of larvae at time 24h shown in Fig. 5, were replotted against the applied bioinsecticide concentration. The resulting graph generated a downward-slopping sigmoidal curve, which its fitting function equation was used to estimate LD50 and LD90 approximately 1.5 × 10^−3^ μg/mL and 1.5 × 10^−2^ μg/mL, respectively.

### Effects of CHSA and B silencing by the bioinsecticide on larval morphology

**Figure 6:**
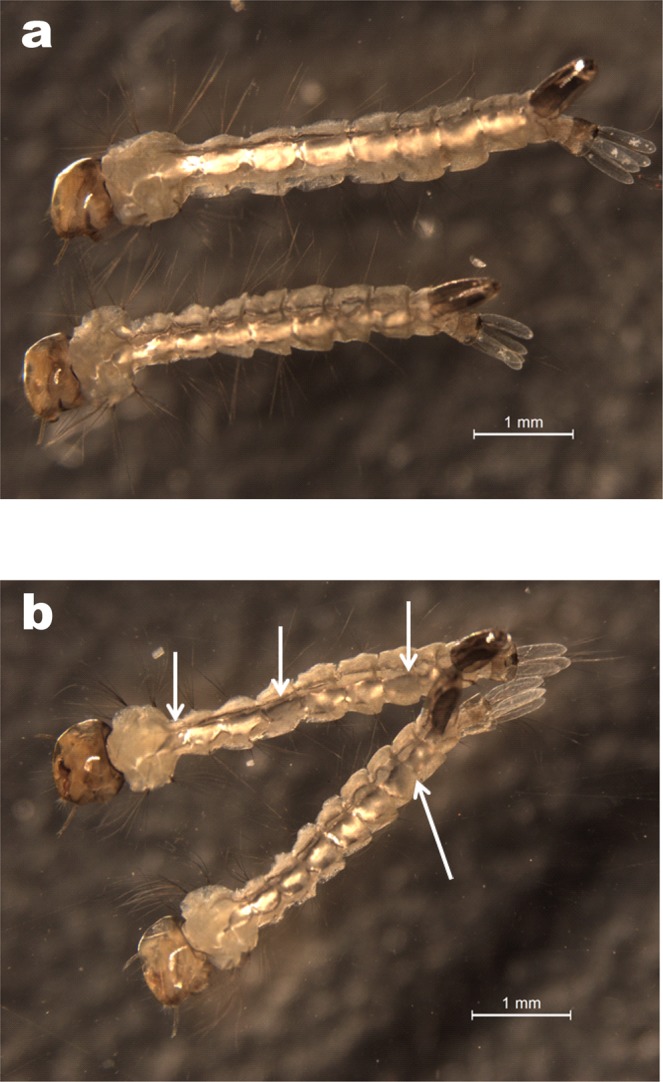
Representative images obtained using optical microscopy of larvae without dsRNA treatment: (**a**) control and (**b**) treated at the 1^st^-instar with 2 × 10^−2^ µg/mL of bioinsecticide. Scale bars = 1 mm. White arrows indicate malformations in larval morphology.

As shown in Fig. 6b, the larvae exhibited morphological differences such as a reduction in the chitinous bristles located on the abdominal segments and the thorax. Additionally, a thinner and more transparent cuticle than that of control larvae, along with holes in the cuticle and an irregular border along the body, were observed in the treated larvae (Fig. 6a). Abnormalities could also be observed throughout the intestine, where there was a reduction and interruption in the light signal that characterizes the intestine. Additionally, a narrowing in the thorax region could be observed. The white arrows indicate malformations of the larval morphology, specifically in the larval abdominal segments. These alterations in the structures of the larvae treated with the bioinsecticide may have been caused by the decreased chitin content, due to the reduced expression of the *CHSA* and *B* genes induced by dsRNA-based gene silencing.

**Figure 7:**
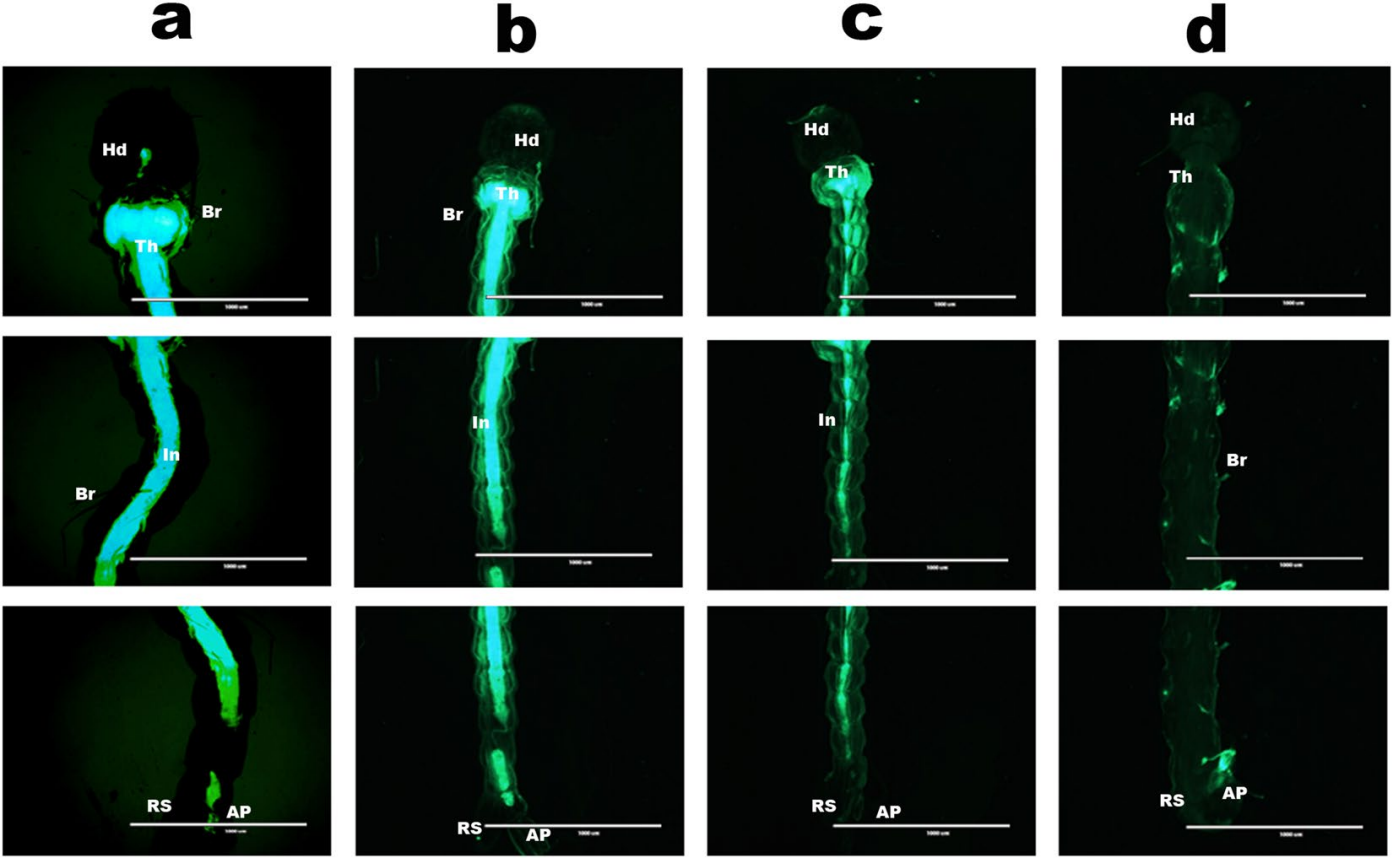
Representative images obtained using fluorescence microscopy of larvae of 4^th^-instar. Larvae of the 1^th^-instar were treated with three different concentrations of bioinsecticide (pCHSA_1928) and analysed by fluorescence microscopy at 4^th^-instar. A FITC-WGA probe was used to detect the chitin polymer, which is indicated by the green FITC-WGA fluorescence signal. Control group without addition of bioinsecticide (**a**) and larvae treated with 2 × 10^−4^ µg/mL (**b**) 2 × 10^−3^ µg/mL (**c**) or 2 × 10^−2^ µg/mL (**d**) of bioinsecticide. In all groups, various segments of the insect body were photographed: head (Hd), thorax (Th), intestine (In), respiratory siphon (RS), chitinous bristles (Br), and anal papillae (AP). Scale bars = 1 mm.

To determine whether the decreases in *CHSA* and *B* gene expression observed by qPCR directly affected chitin synthesis, larvae from all groups were incubated with a specific FITC-WGA probe for the detection of chitin. As shown in Fig. 7a, the 4^th^-instar control larvae presented a strong FITC-WGA fluorescence signal, indicating the presence of chitin in the cuticle and intestine. However, this visible fluorescence signal progressively weakened with increasing bioinsecticide concentration, being weakest in the group treated with the highest dose, i.e., 2 × 10^−2^ μg/mL, of bioinsecticide (Fig. 7b–d). The lateral chitinous bristles (Br) present on the thorax and abdomen were also reduced in number and size in the larvae treated with different concentrations of the bioinsecticide relative to their number/size in the control larvae. The chitin content of the anal papillae (AP) and respiratory siphon (RS) were greatly altered in the bioinsecticide-treated larvae relative to the control larvae. At the highest concentration of bioinsecticide (2 × 10^−2^ μg/mL), these structures seemed to be absent compared to the control, due to a reduction in the chitin content. At a bioinsecticide concentration of 2 × 10^−2^ μg/mL, alterations in the morphologies of the larval thorax (Th), head (Hd), intestine (In) and anal papillae were observed.

Taken together, these results characterize a new potential RNAi-based bioinsecticide that can function alone or in association with insecticides for mosquito control. This bioinsecticide reduces CHSA and B transcript levels, weakening chitinous structures of larvae that are essential for their survival. This bioinsecticide can also effectively kill larvae at different stages of development and cause deformities in adults when administered to mature larvae.

## Discussion

Numerous studies have indicated that RNAi is a powerful tool for controlling insect populations and may be less vulnerable to insect resistance than other strategies^36^. The goal of our work was to develop a fast-acting larvicidal bioinsecticide that can be directly added to breeding water. We used the *E*. *coli*-expressing dsCHSA_1928, with the aim of silencing both the CHSA and B genes. With the development of this approach, the amount of chemical insecticides or growth inhibitor compound used for insect control could be reduced via the addition of the bioinsecticide as an insecticidal adjuvant agent, as demonstrated with DFB (Fig. 2), thereby minimizing the environmental impact. As a proof of concept performed using dsRNA produced *in vitro* to target specific regions of the CHSA and B transcripts, each dsRNA was added directly to water containing L1 larvae, as described by Figueira-Mansur *et al*.^37^ and Singh *et al*.^25^. The larvae treated with any of the dsRNAs exhibited significantly decreased survival rates compared to the control groups (Fig. 1). However, the most significant reduction in the survival rate was induced by dsCHSA_1928. Based on the alignment performed with the CHSA and B nucleotide sequences of *Ae*. *aegypti* (Suppl. Fig. 1), the siRNAs formed through *Dicer* processing could silence both CHS genes simultaneously (Fig. 1b,c). A similar result was obtained by Zhang *et al*.^32^, which can be explained by a mechanism closely mirroring micro RNA-silencing^38^. Comparing Fig. 1a with 1d reveals that dsRNAs produced *in vitro* were more efficient in killing larvae than were *E*. *coli* expressing the dsRNAs, except for pCHSA_1928.

The efficient control of insects by RNAi requires autonomous dsRNA uptake by the insect through dietary ingestion or the epidermis. We added naked dsRNA to the larval rearing water and found similar results in terms of reduced CHSA and B transcript levels (Fig. 1), without a need for a carrier (Zhang *et al*.^32^) or osmotic pressure (López-Martinez *et al*.^39^) for dsRNA uptake. Since dsCHSA_1928 could efficiently kill larvae via gene silencing, we considered a simpler and more cost-effective approach for bioinsecticide application and production, which would enable us to obtain dsRNA in large quantities for insect population control. We used a genetically modified *E*. *coli* bacterium free of RNase III to express dsRNA; a similar approach was applied by Whyard *et al*.^40^ for mosquito population suppression. We chose lactose instead of IPTG for the induction of dsRNA expression to reduce the production cost, as performed by Bashir *et al*.^41^. To rupture the cells and release the dsRNA, we chose a solution of chlorhexidine, a cationic detergent belonging to the bisbiguanide class of compounds^42^, This detergent is more inexpensive, easier to use and more effective than sonication or the bactericidal 70% ethanol methods tested (data not shown)^43^.

The bioinsecticide has an insecticidal adjuvant effect (Fig. 2), probably due to its weakening of the cuticle and intestine of the mosquito, facilitating the penetration of the benzophenylurea (BFU) DFB. Douris *et al*.^44^ used CRISPR/Cas9 technology to show that inhibition occurs through interaction with the CHSA enzyme. Effects of BFU on egg chitin synthesis and other chitinous structures have been demonstrated in other insects^45,46^. The simultaneous action of DFB upon the CHSA enzyme, proposed by Douris *et al*.^44^, and CHSA and B gene knockdown caused by dsCHSA_1928 might explain the enhancement of DFB larvicidal activity observed in the presence of the bioinsecticide. This strategy was also effective in advanced stages of larval development, as shown by its larvicidal activity and decreased CHSA and B transcript levels in 4^th^-instar larvae (Fig. 3). Figure 4a and b show that silencing affected moulting, and the silencing phenotype was maintained until the adult phase, when the emerged adults appeared smaller and had more fragile cuticles than control insects (Fig. 4c). In Diptera, there is still no evidence of a classical mechanism of dsRNA entry and/or amplification involving the genes already characterized in other organisms^26,47,48^. However, numerous reports showing robustness of amplification, phenotype durability and transmission to offspring suggest that these organisms may possess alternative mechanisms for silencing by RNAi that differ from those described in the literature^34,35^. We also tested whether the silencing phenotype was modified in a dose-dependent manner. As expected, survival rates significantly decreased with an increasing bioinsecticide concentration but not in a dose-dependent manner (Fig. 5a). This effect may have occurred because the increase in the bioinsecticide treatment dose did not reduce CHSA and B transcript levels in a dose-dependent manner, probably due to the still-undiscovered amplification mechanism involved in gene silencing in *Diptera* (Fig. 5b,c). A response to RNAi targeting CHSA was also reported in *Locusta migratoria*; the insects exhibited morphological alterations of the wings and reduced size^49^, as observed in the present study (Fig. 4c). The variability in silencing responsiveness and the strength of silencing have been shown to be related to the efficient take-up of dsRNA by cell lines, the processing of dsRNA to siRNA^50^, the length of morphological barriers, dsRNA concentration^26,47^ and differences in the tissue-specific expression levels of the core machinery for RNAi^47,48,51^. Although the observed difference was statistically significant, the reduction in CHSA and B transcript levels did not differ markedly between different treatment concentrations (Fig. 5b,c), probably because the quantity of dsCHSA_1928 present in the different concentrations of bioinsecticide was sufficient for the amplification mechanism, thereby precluding a dose-dependent response. All concentrations of the bioinsecticide used were enough to affect the chitin metabolism of larvae (Figs 6 and 7). The impact of bioinsecticide addition on chitin content, as revealed by the fluorescence assay in the larval cuticle and intestine, was remarkable and was negatively correlated with the bioinsecticide doses (Fig. 7). Several studies have demonstrated that the effect of RNAi is influenced by the original functions of the target genes^47^. Supporting this finding, various authors have reported a stronger response to RNAi targeting genes with essential functions. The CHSA and B enzymes, which belong to the pathways responsible for chitin synthesis in the ectodermal tissues and midgut^20,23,52^, are biologically essential to maintain life in insects, reinforcing their advantages as targets for population control. According to the evidence obtained in this work, chitin metabolism is directly affected by RNAi silencing induction using the pCHSA_1928 bioinsecticide, as shown in Figs 6 and 7. In addition, CHSA transcript levels were quite low, due to silencing induced by the bioinsecticide (Figs 1b,e and 3b). As demonstrated in this work, we have developed an approach that can be adapted to another expression system, such as *Saccharomyces cerevisiae*, as successfully shown by Mysore *et al*.^53^ who used yeast interfering RNA larvicides targeting neural genes to induce mortality. However, further studies are necessary to establish the conditions for expression and large-scale culture and to determine the effectiveness of this bioinsecticide via different forms of administration, such as spraying, in all stages of mosquito development. In addition, the use of this expression system in field applications should be regulated by the appropriate authorities in each country, although, unfortunately, not every country has a regulatory agency. Taken together, the data presented herein indicate that chitin metabolism and, particularly, the double silencing of *CHSA* and *B* genes is a good strategy to avoid the spread of arboviral infections via *Ae*. *aegypti* population control.

## Material and Methods

### Insects and ethics statement

Mosquito maintenance and ethical protocols were performed as described by Figueira-Mansur *et al*.^37^.

### CHS sequence alignments

The amino acid sequence of CHSA from *Tribolium castaneum* (NP_0010394021) was used as a query sequence in blast searches performed against the *Ae*. *aegypti* protein database in VectorBase (https://www.vectorbase.org/)^54^ with the blastp program^55^ using the default settings. Two putative CHS sequences were selected based on showing the highest identity with the query sequence. The homologous nucleotide sequences of the putative *CHS* genes were also acquired, and alignments were performed as described previously^56^. Then, three regions of the CHS A nucleotide sequence, 2718_1 (1550–1750 nt), 2718_2 (1064–1291 nt) and 2718_3 (1928–2114 nt), and two regions of CHS B, 5618_1 (1205–1384 nt) and 5618_2 (693–940 nt), were selected as targets for gene silencing by RNAi.

### *In vitro* and *in vivo* dsRNA synthesis

The target CHS regions were selected, and oligonucleotides were designed to amplify these specific sequences for *in vitro* synthesis of the dsRNAs. For this purpose, the T7 promoter sequence was included. For this step, the IDT online tool was used (https://www.idtdna.com/pages/products/gene-expression/molecular-beacons). The dsRNA molecules were synthesized *in vitro* using the MEGAscript RNAi Kit (Ambion) according to the manufacturer’s instructions. The maltose-binding protein (*MalE*) gene from *Escherichia coli* was used as an unrelated gene control, dsMalE^37^. The gene sequences of the three regions from *CHSA* and two regions from *CHSB* selected above were targeted by the specific synthesized dsRNAs, referred to as dsCHSA_1064, dsCHSA_1550, dsCHSA_1928, dsCHSB_693 and dsCHSB_1205, respectively. The synthesized dsRNAs were quantified according to Lee and Schmittgen^57^, and their integrities were evaluated by electrophoresis in 1% agarose gels. The cDNA fragments of the CHS regions (2718_1, 2718_2, 2718_3, 5618_1 and 5618_2) were cloned into the PL4440 plasmid, which contains two convergent T7 promoters in opposite orientations, to express the dsRNAs using the enzyme T7 polymerase. As an expression system, the *E*. *coli* HT115 (DE3) strain, kindly provided by Minnesota University, was used^58^. PL4440 was a gift from Andrew Fire (Addgene plasmid # 1654). The target sequences of the *CHS* genes were cloned using restriction sites for the *Sma*I (forward) and *Xho*I (reverse) enzymes. A restriction site analysis for cloning was carried out using the NEBcutter online tool (http://www.neb.uk.com). The clones of the different *CHS*-silencing regions in the vector PL4440 were produced by the Epoch Life Science company (Missouri City, Texas). *E*. *coli* HT115 (DE3) competent cells were prepared using the standard calcium chloride method^59^. Bacterial culture and transformation were performed as described by Inoue *et al*.^60^. Double-stranded RNA expression was induced with 1 mM IPTG^56,61^ or 10 g/L lactose^41^. Five hours after induction, the bacterial cells harbouring each CHS construct were harvested by centrifugation, and the pellet was washed with ultrapure water to remove the culture medium residue. The pellet was inactivated with 0.5% chlorhexidine solution, and the bacterial lysate containing dsRNA was used as a bioinsecticide in larvicidal assays.

### Induction of silencing by *in vitro* dsRNA and *in vivo* dsRNA expressed in *E*. *coli* HT115

Groups of thirty 1^st^-instar larvae were treated separately with dsCHSA_1064, dsCHSA_1550, dsCHSB_693, dsCHSB_1205 or dsCHSA_1928 synthesized *in vitro* for silencing. Two control groups, one without dsRNA addition and one with dsMalE (unrelated gene) addition, were also included. Each dsRNA was added directly to breeding water containing 1^st^-instar larvae to a final concentration of 0.2 µg dsRNA/mL of water in a total volume of 2 mL (400 ng dsRNA/2 mL). Twenty-four hours after dsRNA addition, the larvae were fed dog food. The survival rate was recorded until the 4^th^ day after dsRNA addition (Fig. 1). The experiment was performed in biological triplicate. To test the bioinsecticide’s larvicidal effect, 7 groups of 45 larvae in the 1^st^-instar of development were treated with the bioinsecticide (lysate of *E*. *coli* HT115 containing the PL4440 plasmid with each of five inserts, pCHSA_1064, pCHSA_1550, pCHSB_693, pCHSB_1205 and pCHSA_1928, to express dsCHSA_1064, dsCHSA_1550, dsCHSB_693, dsCHSB_1205, and dsCHSA_1928, respectively, in 2 × 10^−2^ µg/mL of lysed cellular suspension (~4000 ng dsRNA/2 mL assay)). The bioinsecticide’s larvicidal effect was verified at an advanced larval stage (4^th^-instar) using the same experimental protocol. Both experiments were performed using three biological replicates. The development of survivors was monitored throughout the life cycle. To verify that the bioinsecticide concentration was correlated with penetrance of the silencing phenotype, three different concentrations (2 × 10^−2^, 2 × 10^−3^ and 2 × 10^−4^ µg/mL) of the pCHSA_1928 bioinsecticide without a carrier (*E*. *coli* lysate containing dsCHSA_1928) were added directly to tap breeding water containing experimental groups of 20 larvae in the 1^st^-instar. This experiment was performed with 5 biological replicates. Controls were established using larval groups without dsRNA addition, larvae with *E*. *coli* HT115 with empty PL4440 plasmid lysed with chlorhexidine (pL4440) at a concentration of 2 × 10^−2^ µg/mL and larvae exposed to *E*. *coli* HT115 lysed with chlorhexidine at a concentration of 2 × 10^−2^ µg/mL. Twenty-four hours after bioinsecticide treatment, the larvae were fed, and mortality rates were recorded daily.

### The bioinsecticide as an insecticidal adjuvant in association with DFB

To test the insecticidal adjuvant effect of the bioinsecticide, a larvicidal assay was performed in association with DFB at a final concentration of 10^−4^ mg/L (LC_50_, previously determined in our laboratory). Five groups of 15 1^st^-instar larvae were subjected to the following treatments for three days: control group, no addition of DFB or bioinsecticide; treatment with only the pCHSA_1928 bioinsecticide at 2 × 10^−2^ µg/mL of cell lysate suspension; treatment with the bioinsecticide (2 × 10^−2^ μg/mL), followed by DFB (10^−4^ mg/L) when the larvae reached the 3^rd^-instar; treatment with only DFB (10^−4^ mg/L) when the larvae reached the 3^rd^-instar; and a diflubenzuron solvent toxicity control group was treated with acetone when the larvae reached the 3^rd^-instar. After the treatments, the larvae were fed, and mortality rates were recorded daily. The experiment was performed in biological triplicate.

### Verification of mRNA knockdown

Total RNA from ten larvae of each biological replicate (for the 1^st^-instar larvae, 10 larvae were used per biological replicate) was extracted using TRIzol Sigma Chemical Co. (St Louis, MO, USA) according to the manufacturer’s instructions. First-strand cDNA synthesis was performed using 1 μg of RNA with a cDNA synthesis kit, employing random primers (Applied Biosystems). The first-strand cDNA obtained was used as a template for quantitative PCR (qPCR), which was performed using a StepOne real-time PCR system (Applied Biosystems) and SYBR^®^ Green PCR Master Mix (Applied Biosystems) following the manufacturer’s instructions. Approximately 50 ng/μL cDNA and gene-specific primers (500 nM) were used for each reaction mixture. Ribosomal protein S6 (RPS6, gene AAEL000032) was used as the reference gene to normalize *CHS* expression levels^37^. The qPCR assays were conducted according to the Minimum Information Required for Publication of Quantitative Real-Time PCR Experiments (MIQE) Guidelines^62^ in biological triplicate. Raw quantification cycle (Cq) values normalized to those of the RPS6 standard were then used to calculate the relative expression levels in the samples using the 2-ΔΔCt method^63^. The expression of the *CHSA* and *B* genes in the control groups, i.e., larvae without dsRNA addition, larvae with *E*. *coli* HT115 with empty PL4440 plasmid lysed with chlorhexidine (pL4440) and larvae with dsMalE addition, was considered the basal level (or 1).

### Phenotype description

After 48 h of bioinsecticide treatment at the highest concentration (2 × 10^−2^ μg/mL), larvae and emerging adults were observed using a stereoscopic microscope (Leica M205 FA) to evaluate the morphological changes associated with the bioinsecticide.

### Microscopy using a fluorescein-5-isothiocyanate-labelled wheat germ agglutinin (FITC-WGA) probe (Sigma-Aldrich)

The surviving larvae from the bioinsecticide treatment were analysed via fluorescence microscopy with a specific FITC-WGA probe for the detection of chitin, as described by Moreira *et al*.^18^. The insects were photographed using a fluorescence stereomicroscope (SZX12, Olympus). Larvae treated with the three bioinsecticide concentrations, 2 × 10^−2^ µg/mL (4000 ng dsRNA/2 mL), 2 × 10^−3^ µg/mL (400 ng dsRNA/2 mL), and 2 × 10^−4^ µg/mL(40 ng dsRNA/2 mL), and larvae from the control group were incubated separately in contact with the solution containing the probe for 1 h and protected from light. The magnification was 4×, and the scale bar was 1 mm. Three separate segments of the insect body were photographed: head, abdomen and anal papillae.

### Data analysis

ANOVA and Tukey’s tests were performed with Prism 6.04 (GraphPad Software). All significant values had p-values less than 0.05.

## Supplementary information

Supplemental Figure 1.
