# Supplementary material for: RNAi-based bioinsecticide for Aedes mosquito control
Source: Sci Rep. 2019 Mar 11;9:4038. doi: 10.1038/s41598-019-39666-5 (PMC6411920; doi:10.1038/s41598-019-39666-5)
Supplement: Supplementary file 1 — Supplemental Figure 1. [file 41598_2019_39666_MOESM1_ESM.pdf]

RNAi-based bioinsecticide for *Aedes* mosquito control

Sheila Barbara G. Lopez[1], Victor Guimarães-Ribeiro[1], João Victor G. Rodriguez[1], Fernando Augusto Dorand[1], Tiago S. Salles[1], Thayane E. Sá-Guimarães[1], Evelyn S. L. Alvarenga[1], Ana Claudia A. Melo[1,2], Rodrigo V. Almeida[1] & Monica F. Moreira[1,2,*]

1. Universidade Federal do Rio de Janeiro, Departamento de Bioquímica, Instituto de Química, 21941-909, Rio de Janeiro, RJ, Brazil.

2. Instituto Nacional de Ciência e Tecnologia em Entomologia Molecular, Brazil.

* Address for correspondence: Prof. Monica Ferreira Moreira, Phone: +55 21 39387365; e-mail: monica@iq.ufrj.br

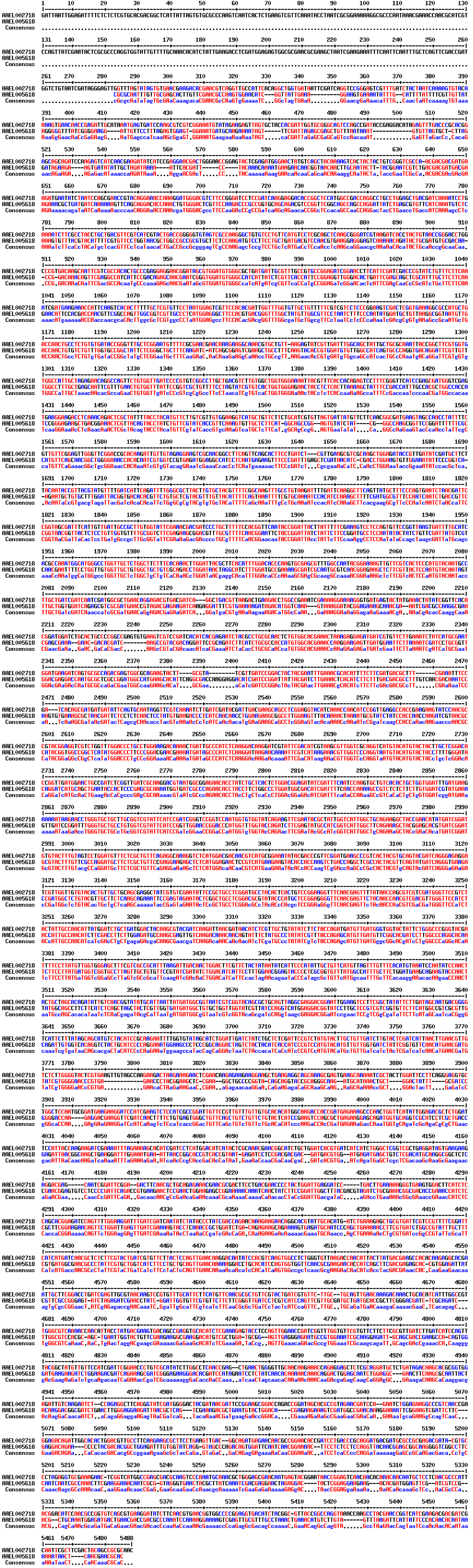
AAEL002718
AAEL005618
Consensus

Supplemental Figure 1. Alignment of AAEL002718 (CHS A) and AAEL005618 (CHSB) nucleotide sequences of the mosquito Ae. aegypti. The regions chosen as targets for gene silencing were as follows: three regions of CHS A target for silencing, i.e., 1550–1750, 1064–1291 and 1928–2114 nt; and two regions of CHS B, i.e., 1205–1384 and 693–940 nt.
